# A digital method for wear volume loss analysis using a single-scan three-dimensional dataset

**DOI:** 10.1016/j.jds.2021.06.015

**Published:** 2021-07-04

**Authors:** Jae-Hyun Lee, Gerelmaa Myagmar, Ho-Beom Kwon, Jung-Suk Han

**Affiliations:** a Department of Prosthodontics and Dental Research Institute, School of Dentistry, Seoul National University, Seoul, South Korea; b MAS Program of Digital Dental Technologies, University Clinics of Dental Medicine, Faculty of Medicine, University of Geneva, Geneva, Switzerland

**Keywords:** Computer-aided design, Dental materials, Digital dentistry, Dental restoration wear, Tooth wear

## Abstract

A straightforward digital method of evaluating wear volume loss is described. This method allows the measurement of the wear by analyzing only the three-dimensional scan dataset of the worn specimen without needing a separate baseline scan. Compared to the conventional method, involving superimposition of the two datasets scanned before and after the wear test, this method can reduce labor and accuracy errors caused by repeated scans and superimposition procedures. Further, this analysis can be conducted using free computer-aided design software, which makes it more efficient for the analysis of wear volume loss of restorative materials.

## Introduction

Most dental restorative materials and natural teeth are subjected to substance loss due to wear during mastication.^1^ Since the difference in wear resistance between the restoration and tooth enamel can cause an overload on the teeth or chewing discomfort, the wear resistance of the restorative material should be similar to that of natural enamel.^2^ In addition, the low wear resistance of the restorative material may lead to thinning of the material and mechanical complications, such as cracks, fractures, and perforations in the restorations.^3^ The wear behavior of newly developed restorative materials is a topic of interest among dental researchers and clinicians.

An in vitro wear test is usually performed by measuring the specimen before and after masticatory simulation and analyzing its wear volume or depth. The wear amount of dental restorations has been investigated using various measurement methods, such as profilometry,^4^ photogrammetry,^5^ and three-dimensional (3D) contact scanning.^2^ Recently, with the development of digital dental technologies, 3D desktop scanners or intraoral scanners have been frequently used for wear analysis.^6^^,^^7^ These 3D dental scanners have the advantage of being highly accessible and familiar to most dental researchers and technicians. In general, wear analysis using these devices involves scanning of a specimen once at baseline before the wear test and then again after simulating mastication.^6^^,^^7^ Subsequently, the difference is calculated by superimposing the 3D dataset before and after the wear test using a high-price 3D inspection software.^6^^,^^7^ Instead of this conventional 3D superimposition method, the present article describes a simple method of calculating the wear volume loss using a free software with only one scan that is performed after the wear test.

## Technique

1. Fabricate the substrate specimen for the wear test. The surface of the specimen to be worn by the abrader must be flat.
2. Conduct a wear test in a conventional manner (Fig. 1A).

**Figure 1:**
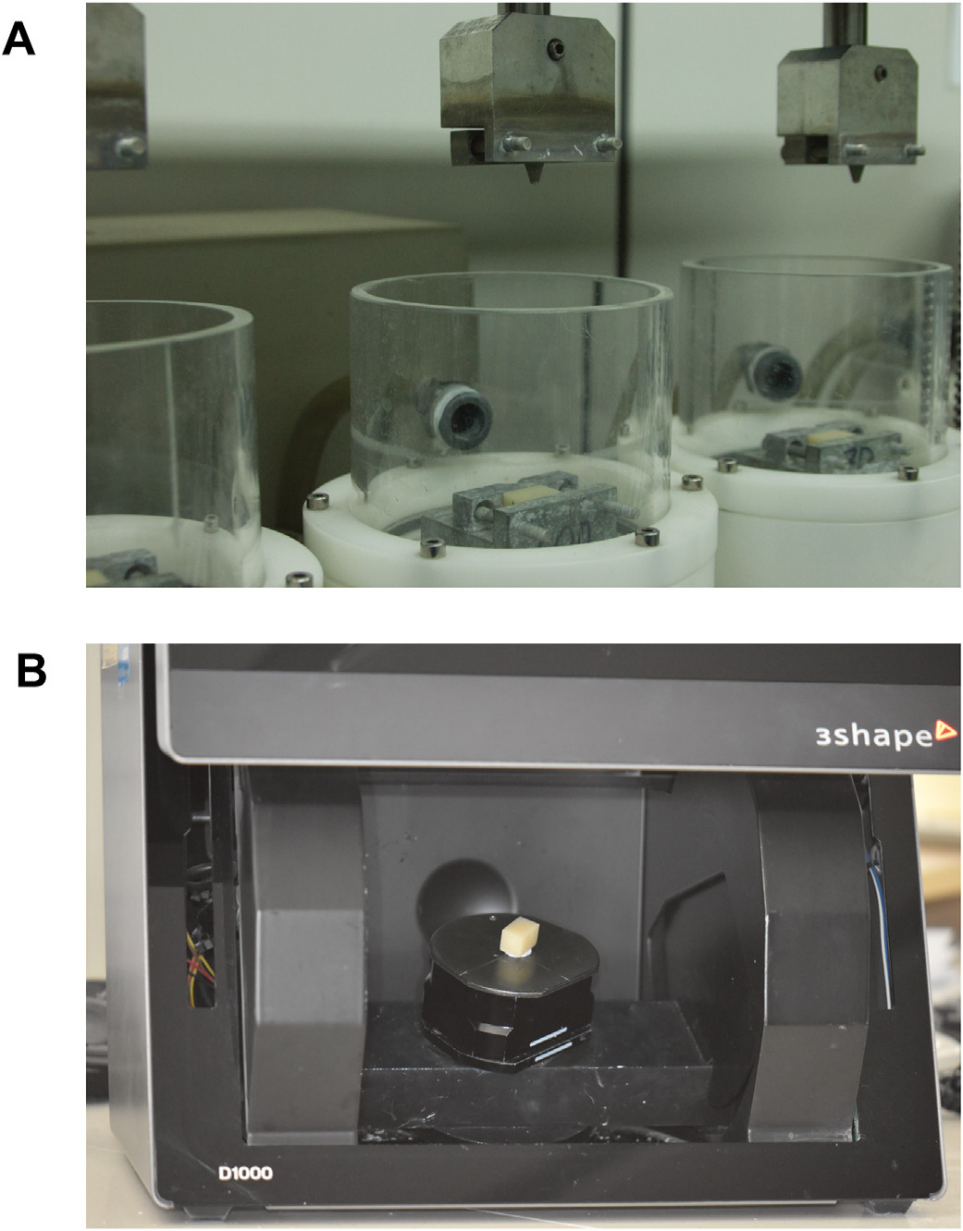
Chewing simulation and digital data acquisition. (A) Chewing simulator with restorative material specimens and abraders. (B) Scanning all surfaces of the worn specimen using a three-dimensional desktop scanner.
3. Scan all surfaces of the worn specimen using a 3D desktop scanner (D1000 desktop scanner; 3Shape A/S, Copenhagen, Denmark) and export the scan dataset in a standard tessellation language (STL) file format (Fig. 1B).
4. Import the STL file into the free computer-aided design (CAD) software program (Autodesk MeshMixer, v3.5.474; Autodesk, Inc., San Rafael, CA, USA).
5. Click on the “Analysis” button on the menu bar and select the “Inspector” function to check for holes in the model. If necessary, click on the “Auto repair all” button to remove the holes.
6. Click on the “Analysis” menu again and select the “Stability” function. Record the current volume of the 3D model (Fig. 2A).

**Figure 2:**
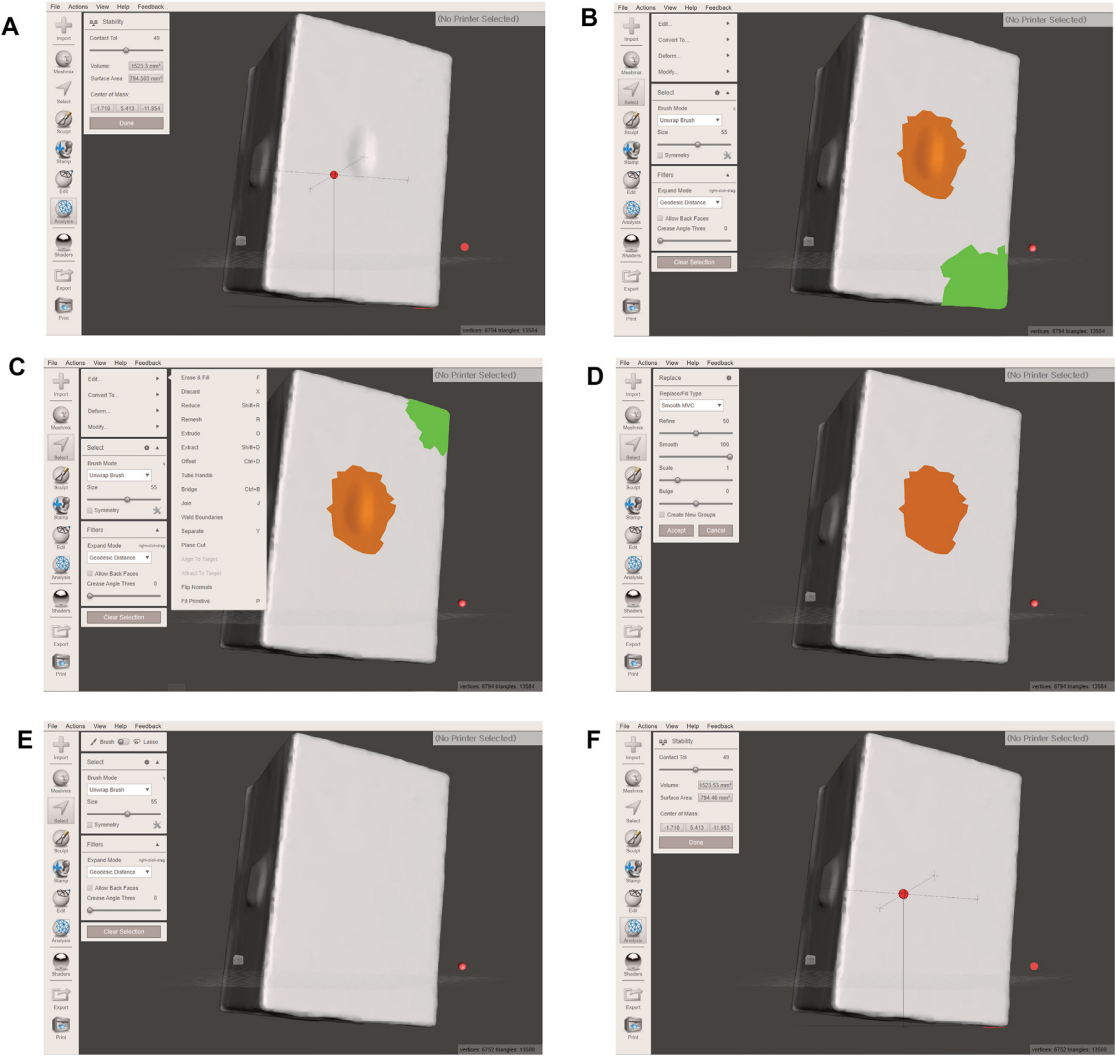
Digital wear analysis using a single-scan three-dimensional dataset. (A) The current volume of the specimen can be seen using the “Stability” function in the “Analysis” menu. The current volume of the specimen as seen in this figure is 1523.3 mm^3^. (B) The worn area of the specimen is indicated using the “Select” menu, seen as the area in orange. The green area indicates the position of the mouse cursor and has no particular meaning in this figure. (C) The worn area is filled by applying the “Erase & Fill” function from the “Edit” menu. The green area indicates the position of the mouse cursor and has no particular meaning in this figure. (D) After clicking on “Accept” in the “Erase & Fill” function. (E) The flattened surface of the specimen is seen, representing its condition before undergoing the wear test. (F) The current volume of the specimen is re-checked for wear volume loss. In this case, the current volume of the specimen is 1523.53 mm^3^. Subtracting the original volume of 1523.3 mm^3^ from this number, the wear volume loss was calculated as 0.23 mm^3^.
7. Using the “Select” menu on the menu bar, select a wide extent of the worn area, including the adjacent non-worn flat surface (Fig. 2B).
8. After selecting the “Edit” option and the “Erase & Fill” function (Fig. 2C), click on “Accept” (Fig. 2D). The worn area will flatten and appear as it was before wearing (Fig. 2E).
9. Use the “Analysis” – “Stability” function again to record the current volume of the model (Fig. 2F).
10. Calculate the wear volume loss by subtracting the original volume from the volume obtained after using the “Erase & Fill” function.

## Discussion

An efficient method to measure the wear volume loss of restorative materials, with only one scan after the wear test and digital analysis, has been described here. The main advantage of this method is that it is possible to evaluate the amount of wear with only one scan after the wear test, without scanning the specimen at baseline for the superimposition process. As the number of scans is reduced, human labor and incidental costs can be lowered. The occurrence of human errors can also be minimized. On the other hand, when using the conventional superimposition method, inaccuracy may increase due to scanners with low repeatability.^8^, ^9^, ^10^ Errors can also occur during the alignment processes for superimposition in the inspection and metrology software.^10^

Another advantage of this method is that it can minimize the cost of additional equipment or software. This analysis method can be conducted using free open-source software instead of proprietary software. In addition, since it can be analyzed using desktop scanners generally available at dental schools, hospitals, and laboratories, no additional equipment is required. Furthermore, a recent study showed the feasibility of using an intraoral scanner, as an alternative to desktop scanners, for wear analysis.^6^ However, the accuracy of intraoral scanners was reported to be relatively poor for flat shapes than for geometric shapes.^8^ Therefore, it would be advantageous to design the other surfaces of the specimen in a more geometric shape, except for the surface planned to be worn, when applying the present described method using an intraoral scanner.^8^

A limitation of this method is that the analysis can only be performed when the flat surface of the specimen is abraded in an in vitro test. It is important to completely flatten the surface that is to be worn before masticatory simulation on the specimen, to reduce errors in the restoration process (“Erase & Fill”) of the CAD software. Therefore, it would be difficult to use this method for the analysis of teeth-shaped specimens or the dentition of human subjects in clinical studies. However, with the ever ongoing development of new materials in the field of restorative dentistry, the described method will be a straightforward and useful tool for in vitro evaluation of the wear resistance of restorative materials. Further studies comparing the experimental results of this method with those of conventional methods will be beneficial to confirm the accuracy and efficiency of this method.

A digital method was used to analyze the wear volume loss of a specimen of a restorative material. This method, which uses only one scan dataset after the wear test, does not require a baseline scan dataset of the specimens. Thus, it can reduce errors occurring due to the low repeatability of the scanner or during the superimposition process of the inspection software. Human errors and labor necessary for repeated specimen scanning can also be minimized. Additionally, because it utilizes a free CAD software, the described technique could be preferable for wear analysis.

## Declaration of competing interest

The authors have no conflicts of interest relevant to this article.
